# How to Keep the Fire Burning: X+Y for the Gen Zs

**DOI:** 10.12688/mep.21052.1

**Published:** 2025-06-25

**Authors:** Christina Chen, Alisa Corrado, Kevin Ratnasamy, Ross E. Myers, Joanna Lewis, Marylouise K. Wilkerson

**Affiliations:** 1 Department of Pediatrics, Rush University Medical Center, Chicago, Illinois, USA; 2 Medical Director, General Pediatrics Unit, Rush University Medical Center, Chicago, Illinois, USA; 3 Associate Program Director, Pediatric Residency Program, Rush University Children's Hospital, Chicago, Illinois, USA; 4 Critical Care Fellow, Department of Pediatrics, Johns Hopkins All Children's Hospital, Saint Petersburg, Florida, USA; 5 Gastroenterology, Hepatology, and Nutrition Fellow, Department of Pediatrics, Stanford University, Palo Alto, California, USA; 6 Department of Pediatrics, Case Western Reserve University School of Medicine, Cleveland, Ohio, USA; 7 Program Director, Pediatric Residency Program, Rainbow Babies & Children’s Hospital, Cleveland, Ohio, USA; 8 Department of Pediatrics, Advocate Children's Hospital - Park Ridge, Park Ridge, Illinois, USA; 9 Program Director, Pediatric Residency Program, Advocate Children’s Hospital – Park Ridge, Park Ridge, Illinois, USA

**Keywords:** X+Y, scheduling, pediatric residency, Generation Z, iGen, medical education

## Abstract

The X+Y scheduling model has become a widely adopted and effective approach within pediatric and internal medicine residency training programs across the United States. A closer examination of the factors contributing to its success reveals the broader societal and generational context influencing today’s medical trainees—specifically, members of Generation Z (Gen Z). This model supports enhanced adaptability and personalization of learning, fosters teamwork and collaboration, integrates flexibility and wellness, and promotes diversity and inclusion—key educational priorities that align closely with Gen Z’s values and learning preferences. Furthermore, the model resonates with and revitalizes foundational principles of adult learning theory. As Gen Z continues to comprise the majority of incoming medical trainees, further research is warranted to evaluate the applicability and effectiveness of the X+Y model in pediatric subspecialty fellowships and other residency programs, particularly in surgical and procedural-based specialties.

## Introduction

### Who is Gen Z?

Generation Z, or “Gen Z,” born approximately between 1995 and 2012 ^1^, comprises the majority of the current graduate medical education trainees. Also known as “iGen,” ^2^ they are characterized by their digital fluency and diverse backgrounds ^3, 4^. Raised amidst the internet and social media boom ^3^, their formative years have also been shaped by pivotal global events including the COVID-19 pandemic and the Black Lives Matter movement. Much like Billy Joel’s 1989 song “We Didn’t Start the Fire” encapsulates the Baby Boomer era, the recent adaptations of Fall Out Boy’s version reflects many of the Gen Z experiences: “bombing Boston Marathon, War on Terror, Sandy Hook, YouTube killed MTV, climate change, self-driving electric cars.” These events, coupled with their digitally-centered, tech-driven identity, influence how they learn and necessitate a reassessment of educational strategies for their medical training ^1, 5– 8^.

At a recent workshop hosted by the Association of Pediatric Program Directors, participants were prompted to share their perceptions of Gen Z learners ^9^. While there was notable appreciation for their technological proficiency, some responses included descriptors such as “whiny,” “poor resilience,” “multi-taskers,” “lazy,” and “separation of career and personal life” ^9 ^. These perceptions underscore a broader conversation about generational differences in the medical field, and examining how these characteristics reflect the upbringing and formative experiences of Gen Z trainees is key to developing strategies to enhance their medical training. By aligning our educational structure with their unique attributes and needs, we can better support their development into effective and resilient physicians.

### What is X+Y scheduling?

The X+Y scheduling model, initially introduced in 2010 as a “4 plus 1 model” in Internal Medicine, was developed to address the ACGME mandate aimed at reducing conflicting inpatient and outpatient responsibilities ^10^. The model was then adopted by pediatric programs through the AIRE (Advancing Innovation in Resident Education) program beginning in 2018 ^11, 12^. Various formats such as “3+1,” “6+2,” and “4+2” exist, each dividing residents into cohorts within their program. Inpatient rotations occur during X weeks while Y weeks integrate ambulatory experiences with their cohort, including continuity and subspecialty clinics in addition to other clinical exposures. Programs that implemented X+Y scheduling reported improved perceived continuity for clinic patients, improved inpatient workflow and handoff quality, and increased teaching opportunities from both residents and faculty ^11, 13 ^. While X+Y scheduling has been extensively studied in internal medicine, pediatrics, and combined medicine-pediatrics residency programs, its application to other specialties, subspecialty fellowships, or international contexts remains limited ^14^.

## Tips for other specialties and programs considering X+Y scheduling

### 1.  Use X+Y scheduling to enhance trainee adaptability and to accommodate for shifting learning styles

Growing up in an age where information, services, and products are accessed instantly, Gen Z learners often exhibit traits of “acquired ADHD,” ^2, 5, 15^ seeking flexibility and immediate feedback akin to their digital experiences ^5^. Although adept at accessing information, they may require guidance in critically evaluating it, and will require this training via engaging ways ^3, 16^. These learners value the personal experiences and discussions of practical applicability that the instructor-student dynamic enables, and they crave “microlearning” opportunities ^5, 6^. X+Y scheduling accommodates these needs by allowing predictable transitions between inpatient and outpatient settings, fostering continuous learning and adaptation. During X weeks devoted to inpatient medicine, residents are no longer rushing to and from clinic and have dedicated time for comprehensive rounds and afternoon discussions, facilitating immediate literature reviews and research based on clinical queries raised during rounds. Longitudinal inpatient exposure, followed by a return to service later in the year, facilitates meaningful, timely feedback and supports the learner’s ongoing implementation of improvements through spaced repetition.

### 2.  Use X+Y scheduling to promote teamwork and collaboration

Despite their digital proficiency, the emphasis on social media, technology and pandemic-induced e-learning has led to Gen Z challenges with interpersonal communication and teamwork ^2, 3, 5^. The cohort-based structure of X+Y scheduling promotes consistent interaction with fellow trainees within stable small groups, enhancing teamwork and fostering deeper relationships and peer collaboration throughout their training. Additionally, the potential for longitudinal curriculum development during Y weeks provides opportunity for sustained small group learning amongst different trainee levels, promoting informal peer teaching and mentorship opportunities.

### 3.  Use X+Y scheduling to support trainee wellness and a healthy work-life integration

With the loss of some interpersonal skills due to technology dependence, as well as constant, free-flowing world-wide news and information through social media, there has been an overall decline in psychosocial wellbeing. Gen Z also seems to be more pragmatic and cautious about the future, thus further necessitating an emphasis on overall wellness in the workplace ^3, 6, 15^.

While this shift has elicited frustration among faculty, the emphasis on fulfilling basic needs as a prerequisite for academic and professional progress is not novel. In the early 20th century, educational theorists moved away from classical behaviorist learning models, embracing more humanistic theories. Maslow’s hierarchy of needs suggests that achieving self-actualization requires first meeting fundamental physiological needs, such as sleep, nutrition, and social stability. Similarly, McCluskey’s Theory of Margin posits that a learner’s capacity for self-improvement (“margin”) depends on the balance between their “power” (resources and protective factors) and “load” (demands placed upon them). Margins outside the optimal range can lead to either boredom or burnout ^17^. This notion of holistic wellness as a foundation for self-actualization predates Gen Z and has been reinvigorated by modern graduate medical education trainees, whose experiences are shaped by global events and evolving technologies that impact their power-to-load ratios ^18 ^. By addressing the fundamental needs outlined in Maslow’s hierarchy, thus optimizing learners’ power and minimizing their load, X+Y scheduling supports Gen Z’s journey toward transformational learning and self-actualization.

In addition, while Gen Z cares less about financial success compared to previous generations, they emphasize the importance of flexibility, personal freedoms, and transparency ^6, 8^. By offering structured Y weeks that include wellness activities and administrative or self-study time, residents can better manage their schedules and foster a healthier work-life balance. Transparent scheduling also promotes peer collaboration and self-managed coverage among residents.

### 4.  Use X+Y scheduling to promote diversity and inclusivity

Gen Z's ethnic diversity and exposure to global issues via streaming platforms has led them to depend on the use of narratives and preference for interactive and inclusive educational models ^4, 6^. X+Y scheduling models accommodate diverse perspectives, fostering global awareness and reflecting the ethnic diversity and social consciousness of this generation. A more predictable clinic schedule allows residents to assist families who may otherwise not have an established medical home or regular pediatrician to ensure regular follow-up appointments. Additionally, the opportunity for longitudinal curriculum development during Y weeks has enabled programs to introduce and expand curricula in areas such as global health, advocacy, and Diversity, Equity, and Inclusion (DEI). A longitudinal curriculum engenders educational opportunities more grounded in reflection, discussion, and narrative medicine surrounding diversity and inclusion in the workplace, healthcare equity, and community health needs.

## Conclusion

While X+Y scheduling echoes andragogical practices initially outlined by 20 ^th^ century educational theorists, it also aligns with the learning preferences and needs of Gen Z residents and addresses many of the operational challenges of medical education today. This educational tool has been well studied in pediatrics and internal medicine residency programs in the U.S., but further research is needed to better understand its feasibility and possible benefits in other specialties, such as surgical residencies which depend on the development of procedural or surgical skills, as well as pediatric subspecialty fellowships. By fostering adaptability, collaboration, and flexibility, X+Y scheduling supports the development of healthcare professionals equipped to meet the evolving challenges of modern healthcare – and allows the fire to keep burning in all of us.

## Data Availability

No data associated with this article.
