# Supplementary material for: How to Keep the Fire Burning: X+Y for the Gen Zs
Source: MedEdPublish (2016). 2025 Oct 15;15:27. Originally published 2025 Jun 25. [Version 2] doi: 10.12688/mep.21052.2 (PMC12455176; doi:10.12688/mep.21052.2)
Supplement: Peer review history

# Reviewer report: Reviewer response for version 2

**DOI:** 10.21956/mep.22858.r43957

**Published:** 2025-10-31

**Authors:** Nazan Karaoğlu

**Affiliations:** 1 Necmettin Erbakan University Faculty of Medicine, Konya, Turkey

I read the reviewed form of the manuscript and satisfied with the corrections. I think it can be indexed now. Kind regards

If evidence from practice is presented, are all the underlying source data available to ensure full reproducibility?

Not applicable

Is the topic of the practical tips discussed accurately in the context of the current literature

Yes

Are all factual statements correct and adequately supported by citations?

Yes

Are the conclusions drawn balanced and justified on the basis of the presented arguments?

Partly

Are arguments sufficiently supported by evidence from the published literature and/or the authors’ practice?

Yes

Reviewer Expertise:

medical education, family medicine, residency training

I confirm that I have read this submission and believe that I have an appropriate level of expertise to confirm that it is of an acceptable scientific standard.

# Reviewer report: Reviewer response for version 2

**DOI:** 10.21956/mep.22858.r43956

**Published:** 2025-10-25

**Authors:** Gianna G. Valenti

**Affiliations:** 1 Children’s Hospital of Michigan/Central Michigan University College of Medicine, Detroit, USA

No further comments.

If evidence from practice is presented, are all the underlying source data available to ensure full reproducibility?

Yes

Is the topic of the practical tips discussed accurately in the context of the current literature

Yes

Are all factual statements correct and adequately supported by citations?

Yes

Are the conclusions drawn balanced and justified on the basis of the presented arguments?

Yes

Are arguments sufficiently supported by evidence from the published literature and/or the authors’ practice?

Yes

Reviewer Expertise:

Medical Education; Pediatric Hematology/Oncology

I confirm that I have read this submission and believe that I have an appropriate level of expertise to confirm that it is of an acceptable scientific standard.

# Reviewer report: Reviewer response for version 1

**DOI:** 10.21956/mep.22551.r42959

**Published:** 2025-09-22

**Authors:** Gianna G. Valenti

**Affiliations:** 1 Children’s Hospital of Michigan/Central Michigan University College of Medicine, Detroit, USA

Summary of the Article:

 This Practical Tips article presents a clear and concise analysis of the X+Y scheduling model as applied to medical education for Generation Z trainees. The authors provide an overview of current generational shifts, emphasizing the unique characteristics of Gen Z, such as their digital fluency, a focus on wellness, and a preference for flexible, diverse, and collaborative learning environments. These generational traits are thoughtfully linked to educational needs and challenges, providing the rationale for adopting the X+Y model. The article explicitly addresses the gap in broader and specialty-specific applications of this model, reflecting on its potential beyond traditional pediatric and internal medicine residency programs.

 The X+Y scheduling model is accurately described, setting the stage for readers unfamiliar with this learning and working framework. The authors trace its roots in internal medicine and its adaptation in pediatrics. The explanation highlights how the model divides trainees into cohorts with dedicated blocks of inpatient and outpatient experiences (e.g., 4+1 weeks), offering predictability and opportunities for focused clinical engagement, enhanced continuity, and improved workflow. This foundational description ensures accessibility for educators new to the concept while stressing practical benefits and educational relevance.

 Overall, this article is an important and timely contribution to medical education literature. It is concise, relevant, and educationally impactful. It raises critical questions about how educational models must evolve to meet the needs of current and future generations of learners. With minor revisions to expand evidence, clarify contrasts with existing practice, and include specific examples of benefits, this paper will serve as a valuable resource for medical educators seeking to innovate scheduling and curricular design to effectively support Generation Z trainees.

 Suggestions for Strengthening the Paper:

- More clearly articulate the comparison between the X+Y model and traditional scheduling (e.g., 1 week of outpatient time vs 0.5-1 day a week) to highlight the strengths and limitations of each. This will help the readers compare the new model to current practice while you are listing the strengths and limitations of the X+Y model.
- Provide concrete examples of the listed benefits (such as improved continuity, workflow, patient handoffs, and increased learning opportunities) to illustrate impact.
- Define “microlearning” opportunities more explicitly.
- Expand the discussion on how the model can address global awareness, social consciousness, and DEI, ensuring a direct link between one-week outpatient blocks and advocacy or opportunities (ie, time to travel for hands-on global health experiences, more dedicated time due to time blocking, etc.)
- Acknowledge potential implementation challenges across other specialties (e.g., adapting X+Y in procedural or surgical specialties, impact on continuity, or institutional logistics) and frame them as areas for further research and innovation.

Is the topic of the practical tips discussed accurately in the context of the current literature?

 Yes: The manuscript provides a thorough synthesis of current literature on both Gen Z learning styles and X+Y scheduling, drawing from a wide array of studies, institutional experiences, and educational theory. The educational shifts and practical challenges for Gen Z trainees are well contextualized, and the discussion aligns with national trends and innovations in graduate medical education.

 Are all factual statements correct and adequately supported by citations?

 Yes: All major factual statements are supported by references. The manuscript cites key examples from practice (e.g., the Association of Pediatric Program Directors workshop) and peer-reviewed articles addressing both scheduling outcomes and generational differences.

 Are arguments sufficiently supported by evidence from the published literature and/or the authors’ practice?

 Yes: The authors integrate findings from the published literature and real-world programmatic experiences, drawing on multi-institutional data and theoretical frameworks such as Maslow’s hierarchy and adult learning theory. Arguments for adaptability, teamwork, wellness, and DEI are substantiated with literature and could be strengthened by providing practical examples.

 If evidence from practice is presented, are all the underlying source data available to ensure full reproducibility?

 Yes: The article outlines the sources for all data discussed. Practical tips are anchored in clearly described sections and referenced across multiple medical settings.

 Are the conclusions drawn balanced and justified on the basis of the presented arguments?

 Yes: The manuscript presents a balanced and justified summary, acknowledging the strengths, limitations, and areas for future research in adapting X+Y scheduling for subspecialties, while also calling for further research to test the adaptability of this model across disciplines.

If evidence from practice is presented, are all the underlying source data available to ensure full reproducibility?

Yes

Is the topic of the practical tips discussed accurately in the context of the current literature

Yes

Are all factual statements correct and adequately supported by citations?

Yes

Are the conclusions drawn balanced and justified on the basis of the presented arguments?

Yes

Are arguments sufficiently supported by evidence from the published literature and/or the authors’ practice?

Yes

Reviewer Expertise:

Medical Education; Pediatric Hematology/Oncology

I confirm that I have read this submission and believe that I have an appropriate level of expertise to confirm that it is of an acceptable scientific standard.

# Response

**Published:** 2025-09-29

**Authors:** Christina Chen

**Affiliations:** Pediatrics, Rush University Medical Center, Chicago, Illinois, USA

Thank you for your thorough review and valuable suggestions.  We have clarified the comparsion between the X+Y scheduling model and traditional scheduling and provided a clear definition of "microlearning."  We also referenced the benefits of the X+Y model as perceived by both residents and faculty.  Given the diverse ways the X+Y model has been applied to support advocacy and DEI, we have incorporated these considerations in a general sense.  Additionally, we included a brief discussion of the potential challenges and limitations of the X+Y scheduling and reframed its applicability to the surgical specialities as an area for furture research rather than a current conclusion.  We sincerely appreciate your feedback, which has helped strengthen this paper an an innovative perspective and useful resource for medical educators.

# Reviewer report: Reviewer response for version 1

**DOI:** 10.21956/mep.22551.r43288

**Published:** 2025-09-12

**Authors:** Nazan Karaoğlu

**Affiliations:** 1 Necmettin Erbakan University Faculty of Medicine, Konya, Turkey

Designing of educational models according to the new generation is an important issue. This article summarizes the practices about x+y schedule in some programs and mainly focuses on the positive effects of it. We get the impression that it also includes the own experiences of the authors because it is in the practical tips section. However, even if there is an experience it is not mentioned. If it is just a literature review i look for the educators perspective who are from the old generations as well as the problems encountered in practice with tips for their solutions.

If evidence from practice is presented, are all the underlying source data available to ensure full reproducibility?

Not applicable

Is the topic of the practical tips discussed accurately in the context of the current literature

Yes

Are all factual statements correct and adequately supported by citations?

Yes

Are the conclusions drawn balanced and justified on the basis of the presented arguments?

Partly

Are arguments sufficiently supported by evidence from the published literature and/or the authors’ practice?

Yes

Reviewer Expertise:

medical education, family medicine, residency training

I confirm that I have read this submission and believe that I have an appropriate level of expertise to confirm that it is of an acceptable scientific standard, however I have significant reservations, as outlined above.

# Response

**Published:** 2025-09-29

**Authors:** Christina Chen

**Affiliations:** Pediatrics, Rush University Medical Center, Chicago, Illinois, USA

We appreciate your recognition of the importance of designing educational models for the new generation of trainees.  In response to your feedback, we revised the tips section to incorporate some of our own perspectives and experiences, adding a more personal dimenion to the practical recommendations.  We also addressed your request for insights from educators of older generations by discussing the challenges they may face in transitioning to X+Y scheduling and offering strategies to help ovecome them.  We believe these additions strengthen the relevance and applicability of the article, and we apprciate your thoughtful suggestions.

# Reviewer report: Reviewer response for version 1

**DOI:** 10.21956/mep.22551.r43287

**Published:** 2025-09-08

**Authors:** Ahmet Ziya Birbilen

**Affiliations:** 1 Pediatric, Hacettepe University Faculty of Medicine Department of Child Health and Diseases, Ankara, Turkey

Reading your paper was both enjoyable and informative for me. Thanks for this chance.

 Your manuscript provides a timely and valuable contribution by exploring the relationship between X+Y scheduling and Generation Z learners. Its clear structure, strong connection with adult learning theories, and practical recommendations are important strengths. 

 To further strengthen the paper, I would encourage adding more recent and direct references from graduate medical education literature to support your arguments about Gen Z characteristics and the outcomes of X+Y scheduling. Including a brief discussion of potential challenges or limitations would also add balance and depth. While it is acceptable that no original data are presented, explicitly stating that the article is a narrative review/commentary would improve clarity for readers. Finally, reframing the applicability to surgical specialties as an area for future research rather than a current conclusion would enhance the scientific rigor of the discussion.

 With these additions, I believe your manuscript will become even stronger, more comprehensive, and impactful.

If evidence from practice is presented, are all the underlying source data available to ensure full reproducibility?

Not applicable

Is the topic of the practical tips discussed accurately in the context of the current literature

Yes

Are all factual statements correct and adequately supported by citations?

Yes

Are the conclusions drawn balanced and justified on the basis of the presented arguments?

Yes

Are arguments sufficiently supported by evidence from the published literature and/or the authors’ practice?

Partly

Reviewer Expertise:

Pediatric emergency, simulation in healthcare

I confirm that I have read this submission and believe that I have an appropriate level of expertise to confirm that it is of an acceptable scientific standard.

# Response

**Published:** 2025-09-29

**Authors:** Christina Chen

**Affiliations:** Pediatrics, Rush University Medical Center, Chicago, Illinois, USA

Thank you for your thoughtful feedback.  We are pleased to hear that you found the article both enjoyable and informative.  In response to your suggestions, we have incorporated more recent references from the graduate medical education literature to further support our discussion of Gen Z characteristics and the outcomes of X+Y scheduling.  To improve clarity for readers, we explicitly noted that the article is structured as a narrative review, which we agree helps set appropriate expectations. Additionally, we included a brief discussion of the potential challenges and limitations associated with X+Y scheduling, and we reframed its applicability to surgical specialities as an area for future reserach rather than a current conclusion.  We believe these revisions provide a more balanced perspective and enhance the depth of our analysis.  We sincerely appreciate your insightful feedback and the opportunity to strengthen our work.
